# Recurrence-associated gene signature in patients with stage I non-small-cell lung cancer

**DOI:** 10.1038/s41598-021-99197-w

**Published:** 2021-10-01

**Authors:** Su Han Cho, Shinkyo Yoon, Dae Ho Lee, Sang-We Kim, Kwoneel Kim

**Affiliations:** 1 Department of Life and Nanopharmaceutical Sciences, Kyung Hee University, Seoul, Republic of Korea; 2 Department of Oncology, Asan Medical Center, University of Ulsan College of Medicine, Seoul, Republic of Korea; 3 Department of Biology, Kyung Hee University, Seoul, Republic of Korea

**Keywords:** Cancer, Cancer genetics, Cancer genomics, Lung cancer

## Abstract

Recurrent gene mutations and fusions in cancer patients are likely to be associated with cancer progression or recurrence by Vogelstein *et al. *(*Science (80-)* **340**, 1546–1558 (2013)). In this study, we investigated gene mutations and fusions that recurrently occurred in early-stage cancer patients with stage I non-small-cell cancer (NSCLC). Targeted exome sequencing was performed to profile the variants and confirmed their fidelity at the gene and pathway levels through comparison with data for stage I lung cancer patients, which was obtained from The Cancer Genome Atlas (TCGA). Next, we identified prognostic gene mutations (ATR, ERBB3, KDR, and MUC6), fusions (GOPC-ROS1 and NTRK1-SH2D2A), and VEGF signaling pathway associated with cancer recurrence. To infer the functional implication of the recurrent variants in early-stage cancers, the extent of their selection pattern was investigated, and they were shown to be under positive selection, implying a selective advantage for cancer progression. Specifically, high selection scores were observed in the variants with significantly high risks for recurrence. Taken together, the results of this study enabled us to identify recurrent gene mutations and fusions in a stage I NSCLC cohort and to demonstrate positive selection, which had implications regarding cancer recurrence.

## Introduction

Lung cancer remains the leading cause of cancer death worldwide. Non-small-cell lung cancer (NSCLC) accounts for 80% of all lung cancers, including squamous-cell carcinoma, adenocarcinoma and large-cell carcinoma. Approximately 30% of NSCLC patients are diagnosed at stage I (pT1a ~ c and N0M0), and the standard treatment of stage I NSCLC is complete surgical resection. Even with complete resection of the tumor, approximately 13% of stage I NSCLC patients eventually experience recurrence^1,2^. Considering the dismal prognosis of recurrent NSCLC, there is an unmet need for further research on the predictive factors of recurrence.

Advances in the understanding of genetic alterations of lung cancer have enabled the molecular subclassification of NSCLC. With the identification of mutually exclusive oncogenic drivers, molecularly targeted agents targeting EGFR, ALK, ROS1, BRAF, and NTRK have become the standard of care in patients with recurrent or metastatic NSCLC^3–7^. For an optimal treatment approach based on individual genetic characteristics, actionable mutation screening using next-generation sequencing (NGS) is considered part of routine clinical practice. NGS enables a “one-size-fits-all approach”, which enables the discovery of rare genetic aberrations and tailoring personalized treatment. Although the time/cost-effectiveness and comprehensiveness of NGS tests have provided a paradigm shift in precision medicine for lung cancer, molecular epidemiological observations exhibit difficulties in addressing true drivers and passenger mutations. Considering that the process of molecular evolution may occur during cancer progression, stage I NSCLC, which consists of only a tumor mass in the lung parenchyma without lymph node metastasis, may represent an ideal model for exploration of the genetic landscape of lung cancer. Thus, we attempted to explore the genomic landscape of stage I NSCLC and to investigate the predictive genetic properties for recurrence in patients with stage I NSCLC.

## Results

### Baseline characteristics and survival data

We enrolled 141 patients with NSCLC, and their baseline characteristics are summarized in Table 1. The median age was 66 years (range 38–82 years), 75 patients (53.2%) were male, and 66 patients (46.8%) were female. The entire study population was Asian. With a median follow-up duration of 35.4 months (range, 7.59–142.8), the median recurrence-free survival was 65.0 months (range, 55.1–74.8). There were 28 recurrence events out of 141 patients.

**Table 1:** Clinical information of 141 NSCLC patients.

Characteristics	Total N = 141	%
Age (median, range, in years)	66 (38–82)	
**Sex**		
Male	75	53.2
Female	66	46.8
**ECOG PS at diagnosis**		
0	56	39.7
1	82	58.2
2–3	3	2.1
**Smoking history**		
Never smoker	74	52.5
Ex-smoker	64	43.3
Current smoker	3	2.1
**Histology**		
Squamous	13	9.2
Adenocarconma	118	83.7
Others	10	7.1
**Surgery**		
Wedge resection	17	12.1
Segementectomy	26	18.4
Lobectomy	94	66.7
Bilobectomy	4	2.8

*ECOG PS* Easter Cooperative Oncology Group Performance Status.

### Landscape of gene mutations and fusions in stage I NSCLC patients

**Figure 1:**
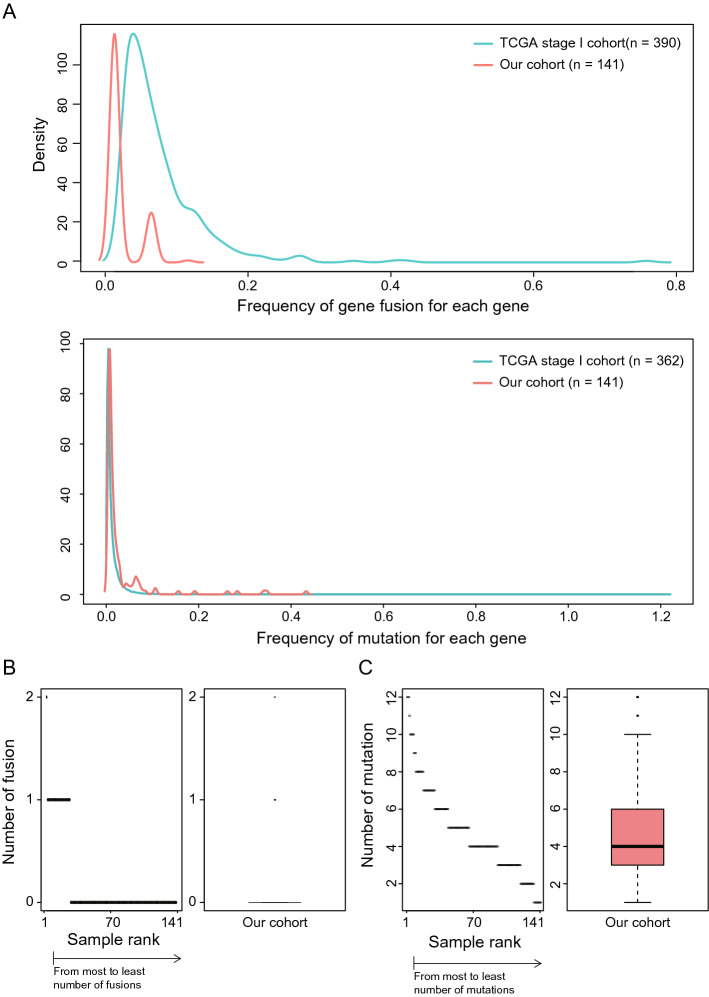
Comparison of the frequency distribution of gene mutations and fusions between our NSCLC cohort and TCGA cohort. (**A**) All fusion genes and mutations were identified from our cohort and TCGA cohort according to the same variant calling procedures (top). Detailed distribution of fusion genes in our cohort (bottom). (**B**) The number of fusion genes was described accordingly by the sample rank that was sorted from most to fewest fusions (left), and the distribution of the number of fusion genes is shown by a box plot (right). (**C**) Detailed distribution of mutations in our cohort. The number of mutations was described according to sample rank that was sorted from most to fewest mutations (left), and the distribution of the number of mutations is shown by a box plot (right). Only the samples that had at least one variant were analyzed for their distributions.

We performed targeted exome-seq covering 323 cancer genes for 650 stage I non-NSCLC patients to profile the landscape of gene mutations and fusions. Detecting gene fusion was highly dependent on the processing of mapped reads. Thus, a variety of process parameters were tested through comparison with the fusion genes reported previously in the TCGA pancancer cohort^8^. The occurrence of gene fusion events in our cohort was similar to that in the TCGA cohort after adjusting the sample size (Fig. 1A, top, r = 0.923; Pearson’s correlation coefficient). Two fusion genes were observed in one patient as most, and more than half of patients (115/141, 82%) were determined to have no fusion gene events (Fig. 1B). We also profiled somatic mutations and their distribution accordingly by each patient and gene. After normalization by total sample size, as described in the fusion gene analysis, a similar distribution pattern of mutations was observed compared with the TCGA cohort (Fig. 1A, bottom, r = 0.949; Pearson’s correlation coefficient). Most patients (133/141, 94%) had more than 2 mutations, and the median mutation frequency was 4 (Fig. 1C, left).

### Recurrent mutations and fusion genes of stage I NSCLC patients and comparison with the TCGA cohort

**Figure 2:**
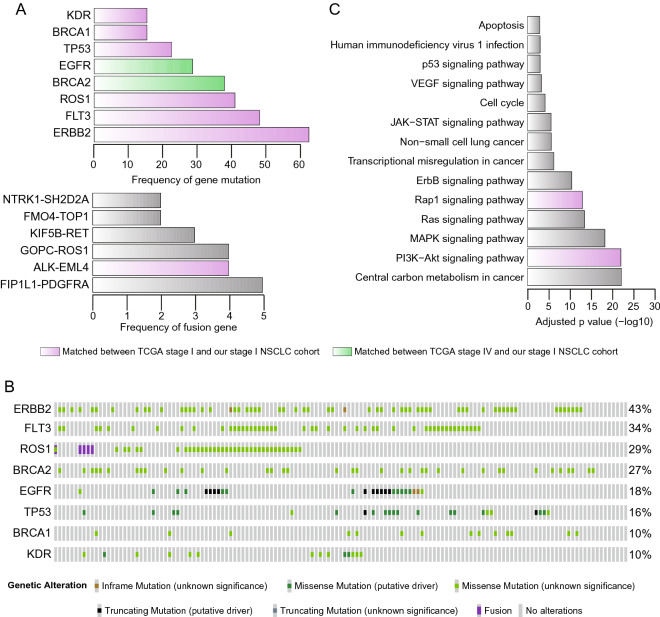
Landscape of gene mutations and fusions. (**A**) The landscape of gene mutations (top) and fusions (bottom) is shown by a bar plot according to their occurrence frequency. Gene mutations with frequencies over 10% and fusion genes with frequencies over two are shown. (**B**) The detailed landscape of gene mutations was described by the OncoPrinter tool^35^. The bars colored gray, purple, and green indicate genes for which variants were reported in none, stage I, and stage IV of the TCGA cohort, respectively. (**C**) Functional enrichment in biological pathways analyzed by EnrichR^30^. The letters colored gray, purple, and green indicate pathways that were enriched in none, stage I, and stage IV of the TCGA cohort, respectively.

Mutations and fusion genes occurring in more than one patient were identified in our stage I NSCLC cohort. We discovered 72 mutations and 6 fusion genes whose frequency was greater than two. There were 8 genes that were frequently mutated by over 10% across our cohort (Fig. 2A). EGFR showed a higher frequency of truncating mutations compared with other genes predominantly exhibiting missense mutations. ROS1 had variants of both missense mutations and gene fusions (Fig. 2B). Three of these variants (KDR, EGFR, and TP53) were known to be significantly mutated or associated with key pathways in lung cancer^9^. We further compared the recurrently mutated genes in our cohort with TCGA early-stage (stage I) and late-stage (stage IV) lung cancer cohorts. Notably, the recurrently mutated genes of the TCGA stage I cohort were more closely matched to our cohort than to the TCGA stage IV cohort (Fig. 2A, top). We investigated how much the genes of each stage were significantly matched to our identified genes by permutation test. As a result, the recurrently mutated genes of our cohort matched more significantly to the recurrently mutated genes of the TCGA stage I cohort than to those of the TCGA stage IV cohort (Supplementary Fig. 1). We obtained 6 recurrent fusion events from our analysis, which did not share any genes with one another; therefore, the events consisted of 12 genes. ALK-EML4 fusion showed the second highest frequency of 2.8% (4/141), which was reported in a previous study^8^ of a TCGA stage I lung cancer cohort (LUAD, frequency was 1.0%). In particular, the recurrent fusion genes of TCGA stage IV lung cancer patients did not overlap with the fusion genes of our cohort (Fig. 2A, bottom). The rest of the fusion events, except ALK-EML4 and KIF5B-RET, had not been reported previously, whereas several genes were known to play a role in lung cancer development when considering the gene level separately. We further investigated how the recurrent mutations and fusion genes were enriched in biological pathways (Fig. 2C). The pathway terms for the retrieved genes were enriched for cancer-related biological functions. These pathway enrichment results were compared with the pathway enrichment findings for TCGA early- and late-stage lung cancer cohorts. As a result, the Rap1 signaling pathway and the PI3K-Akt signaling pathway, which were enriched in our cohort, were also specifically enriched in the TCGA stage I cohort (Fig. 2C). In contrast, the pathways of the TCGA stage IV cohort were not shown to be concordant with the pathways of our cohort.

### Gene mutations and fusions correlated with recurrence in stage I NSCLC patients

**Figure 3:**
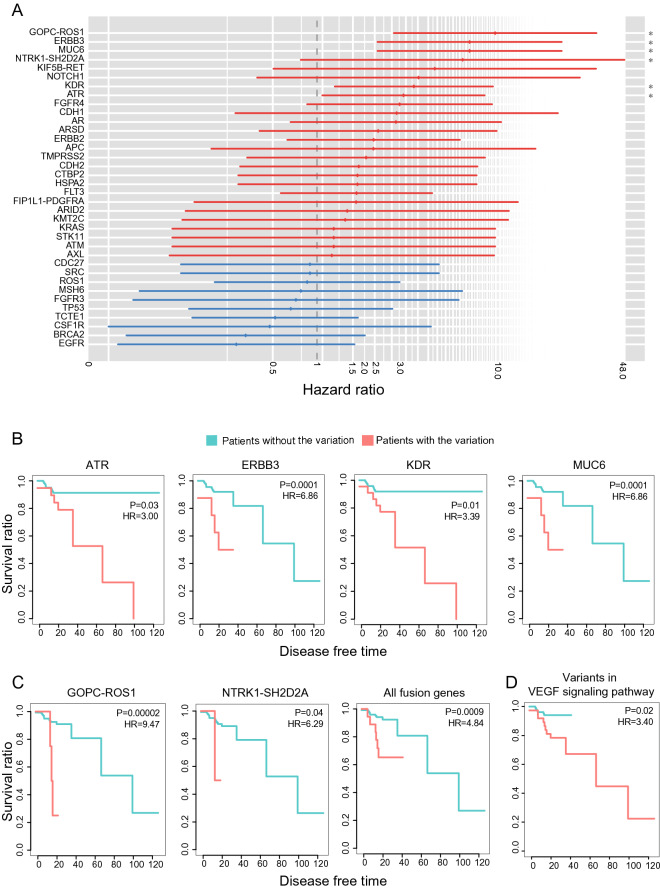
Prognostic signatures for the tumor recurrence. (**A**) The hazard ratio and significance of recurrence-free survival (RFS) tests for gene mutations and fusions in our NSCLC cohort. Only the genes whose frequency was over two were subjected to the RFS test, as it was calculated on a sufficient sample scale. The genes colored red indicate that patients with the gene variant showed a risk of tumor recurrence when compared to patients without the variant. Only the gene marked by an asterisk on the right side showed statistical significance in the RFS test. (**B**) Kaplan–Meier curves of the RFS tests for 4 gene mutations showing a significant hazard ratio. (**C**) Kaplan–Meier curves of the RFS tests for 2 fusion genes showing a significant hazard ratio. All fusion events, including the above two fusion genes, were also assessed by RFS tests. (**D)** RFS tests for the gene set of the VEGF signaling pathway. We combined the status of the variants when they were enriched in a specific cancer-related pathway together when their frequency was more than two. Only the VEGF signaling pathway showed statistical significance in the RFS test among the cancer-related pathways. KDR, KRAS, SRC, and SH2D2A were included in the VEGF signaling pathway with variants in our cohort.

We investigated whether there were signatures of gene mutations or fusions associated with recurrence. Genomic alteration in the early stage could be crucial in driving cancer recurrence because many variants in the later stage have a strong possibility of being passenger mutations due to cancer genome instability. The 32 somatic mutations and 4 fusion genes that recurrently occurred in our stage I NSCLC cohort were selected to analyze recurrence-free survival (RFS). Twenty-two somatic mutations and 4 fusion genes were identified to increase the risk of recurrence when a patient had the variants, but only 4 of the mutated genes and 2 of the fusion genes had a significant correlation with RFS (Fig. 3A–C). The mutation status of ATR, ERBB3, KDR, and MUC6 was significantly associated with patient recurrence, although their frequencies were not notably high; specifically, they were 9 (6.3%) for ATR, 4 (2.8%) for ERBB3, 14 (10%) for KDR, and 2 (1.4%) for MUC6. The second and fourth most frequent fusion genes, GOPC-ROS1 and NTRK1-SH2D2A, showed a significantly high risk of recurrence in the patient group with those fusion events. There were many notable mutations that showed a low occurrence frequency in our cohort. Therefore, we performed RFS analysis for those mutations with an extended gene set that is associated with a relevant pathway. The gene set with VEGF signaling pathway enrichment in our mutation profiling was tested, and their mutation status showed a significant correlation with cancer recurrence (Fig. 3D). KDR, SH2D2A, SRC, and KRAS were included in the VEGF signaling pathway, of which variants were associated with cancer recurrence when they were assessed simultaneously. These 4 genes strongly interacted with each other in physical interactions, cell signaling pathways, and genetic interactions (Supplementary Fig. 2A and B). To examine whether the 4 genes of the VEGF pathway independently contribute to cancer recurrence, the mutual exclusivity of variant occurrence among those 4 genes was profiled. Only one case of coexistence of KDR and KRAS mutations was observed in the same patient, but no cases of concurrence were observed (Supplementary Fig. 2C). We further assessed complementary recurrence patterns of interacting 4 genes, as described in the previous method^10^, for each pair of genes. We calculated variant complementarity, which is the frequency of variants for the gene set of interacting pairs. As shown in Supplementary Fig. 2D, the results showed higher variant complementarity for the interacting gene pairs than all gene pairs, despite low significance. This finding implies that the variants in the 4 genes of the VEGF signaling pathway have sufficient functional potential to be related to cancer recurrence.

### Selective functional implications of gene mutations and fusions associated with cancer recurrence

**Figure 4:**
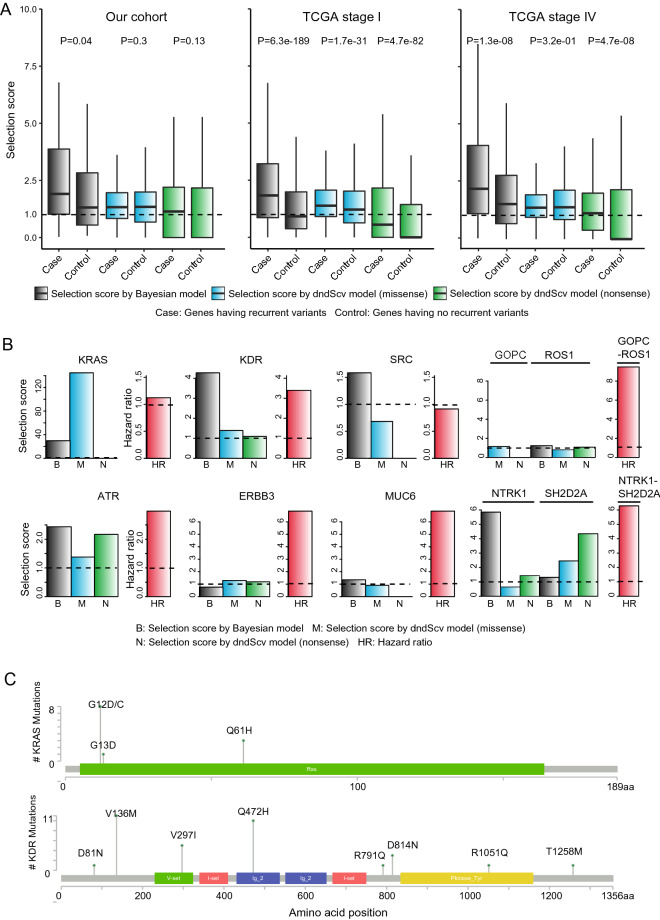
Selective signatures of recurrent gene mutations and fusions. (**A**) Selection values were calculated based on the Bayesian inference and covariate model (*dNdScv*) for the genes having variants of which frequency was over two. The genes with recurrent variants were compared to the genes with no recurrent variants within our cohort (left), stage I TCGA samples (middle), and stage IV TCGA samples (right). The gene numbers that were analyzed by Bayesian inference were 80, 4567, and 256 for our cohort, TCGA stage I samples, and TCGA stage IV samples, respectively. The gene numbers that were analyzed for the selection values by the covariate model were 89, 6863, and 1646 for our cohort, TCGA stage I samples, and TCGA stage IV samples, respectively. (**B**) Selection values for the genes that had a significant association with recurrence-free survival were described. Shown are the selection values obtained for lung adenocarcinoma (LUAD) samples from TCGA. dNdScvM is the normalized ratio of nonsynonymous to synonymous mutations (dN/dS) for missense mutations. (**C**) Genomic structure with amino acid position was described by using MutationMapper tool^35^ for KRAS and KDR. The frequency of each amino acid mutation is shown according to its position.

To infer the functional implication of the recurrent variants identified from our cohort, we examined whether the recurrent gene mutations and fusions had a pattern of positive selection. Recently, selection patterns across a large number of cancer patients have been identified^11,12^ by calculating the ratio of nonsynonymous to synonymous mutations at the gene level. A significantly high frequency of nonsynonymous mutations will be observed for the given background mutation rates of synonymous mutations when they are under positive selection. Thus, this concept can be used to discover candidate genes having cancer driver mutations in addition to passenger mutations. We obtained the selection scores that were previously calculated for each gene by Bayesian inference^11^ and a statistical model for covariates (dNdScv)^12^ based on the mutation patterns observed in the TCGA data. Using these scores, we examined the degree of positive selection on the genes that were recurrently mutated or fused. As a result, both Bayesian inference^11^ and *dNdScv*^12^ indicated positive selection on recurrent gene mutations and fusions in contrast to the absence of recurrent gene mutations and fusions in our cohort (Fig. 4A, left). These signatures were validated by a simulation in which the same number of recurrently mutated genes was tested with permutation (Supplementary Fig. 3). We also calculated the selection score for the recurrently mutated genes in the TCGA early- (stage I) and late-stage (stage IV) lung cancer cohorts. The selection score difference between recurrently and non-recurrently mutated genes was consistent with the difference observed in our cohort, as recurrently mutated genes were under more positive selection than non-recurrently mutated genes (Fig. 4A and Supplementary Fig. 4A, middle and right). Furthermore, higher selective scores in recurrently mutated genes were observed to be more significant for stage I than stage IV, regardless of the measurement method in the permutation test (Supplementary Fig. 3). Taken together, these results indicated that mutations and fusions in the early tumor stage tended to be more subject to positive selection so that they have more functional implications in cancer progression. In our study of tumor recurrence, a significant correlation with RFS was observed in gene mutations (ATR, ERBB3, KDR, and MUC6) and fusions (GOPC-ROS1 and NTRK1-SH2D2A). Interestingly, this significant RFS benefit with two gene mutations (ERBB3 and KDR) was only observed in our early-stage cohort when compared with the TCGA stage IV cohort (Supplementary Fig. 5). While the role of ERBB3 mutation in cancer progression and therapeutic resistance has been previously studied^13,14^, KRAS^15,16^, KDR^17^, and ATR^18,19^ mutations and the GOPC-ROS1^20,21^ fusion gene have also been widely studied to determine their functions in cancer biology. Therefore, we investigated whether the above genes were under positive selection in lung cancer patients (Fig. 4B and Supplementary Fig. 4B). As we expected, most genes showed a selection score over 1, indicating positive selection. Specifically, the Q61H mutation on KRAS, of which the occurrence was 3 in our cohort, was reported for its prognostic and predictive value in advanced NSCLC^22^ and its role as a predictor of resistance to treatment with tyrosine kinase inhibitors in advanced NSCLC^23^ (Fig. 4C). The Q12D/C mutation, whose occurrence was 8 in our cohort, is also well-known for its oncogenic role in proliferation and widespread neoplastic and developmental defects in lung cancer^24^ (Fig. 4C, top). In addition, the Q472H mutation in KDR, whose frequency was 11 in our cohort, has been reported to occur in tyrosine kinase inhibitor-resistant NSCLCs. These previous studies describing the variants in our cohort have implications of oncogenic relevance to lung cancer recurrence.

## Discussion

We profiled one of the largest selective functional studies including only stage I NSCLC patients in an Asian population. Gene mutations and fusions were analyzed by targeted exome-seq (panel-seq), which covered 323 curated genes. Despite the limited resolution of targeted sequencing, this method was employed successfully to identify the genes reported in the previous study for a large lung cancer cohort (TCGA); these genes were KDR, BRCA1, BRCA2, EGFR, ERBB2, and TP53 for mutations and ALK-EML4 and KIF5B-RET for gene fusions. These gene mutations and fusions were compared to the TCGA lung cancer cohort according to stage. We confirmed that a set of recurrent gene mutations and fusions in our cohort was successfully reproduced in the TCGA stage I cohort, rather than the stage IV cohort, at the gene and pathway levels, thereby validating the reliability of our analysis. Furthermore, we investigated whether the recurrently mutated genes are under positive selection as an indicator of cancer progression. The identified genes that occurred recurrently in our cohort were under more positive selection than non-recurrently mutated genes, and the difference was more significant in early-stage lung cancer patients in the TCGA cohort. This result indicates that the genetic variants identified in early-stage cancer by targeted sequencing are able to detect candidate drivers for cancer progression.

Our primary concern was to unravel the signature related to cancer recurrence in stage I lung cancer patients. Four gene mutations (ATR, ERBB3, KDR and MUC6) and two gene fusions (GOPC-ROS1 and NTRK1-SH2D2A) were obtained as markers that had a significant correlation with RFS. Little is known regarding the implications of the identified variants in lung cancer recurrence, except for ERBB3. The feature of positive selection for the variants may also be a “proxy signature” of cancer recurrence because gene mutations and fusions would likely be under positive selection if they brought gain-of-function in cancer recurrence. The identified markers substantially exhibited the signature of positive selection, as expected. In the comparison between early- and late-stage cancer patients in the TCGA cohort, the genes detected in the early stage exhibited higher selection scores. These results imply that genetic variants in the early cancer stage have higher probabilities of cancer recurrence. Clearly, all these findings need to be confirmed by data from larger cohorts. We further attempted to identify the signature associated with cancer recurrence at the system level to overcome limited detection power due to the small sample size. The combinatorial mutation status of the genes in the VEGF signaling pathway showed a significant correlation with cancer recurrence in RFS analysis. In conclusion, recurrent gene mutations and fusions in stage I NSCLC patients have functional potential in cancer progression and recurrence, which we were able to identify with high fidelity through targeted sequencing.

## Materials and methods

### Stage I NSCLC patients

A total of 141 NSCLC patients with surgically resected primary lung cancer were prospectively enrolled from Asan Medical Center between 2009 and December 2018. All patients provided prior written informed consent, and this study was conducted with the approval of the Institutional Review Board of Asan Medical Center. All research was performed in accordance with relevant guidelines/regulations, informed consent was obtained from all participants and/or their legal guardians. The clinicopathological data and survival outcomes were collected prospectively. Surgical tumor tissue sections (at least 4 µm thick) were collected for next-generation sequencing analysis.

### Detection of mutations and fusion genes

Targeted exome-seq was performed with OncoPanel AMC version 4, which was designed by Asan Medical Center through SureDesign. The OncoPanel was composed of 225 genes for entire exons, 6 genes for rearrangements, and 99 hotspots. The sequencing reads were aligned to the hg19 reference genome by using Burrows-Wheeler Aligner (BWA) (http://bio-bwa.sourceforge.net). ^25^. The Genome Analysis Toolkit (GATK) (https://software.broadinstitute.org/gatk). ^26^ was applied for base quality score recalibration, indel realignment, and duplicate removal. The BAM files produced after these processes were subjected to MuTect (https://software.broadinstitute.org/cancer/cga/mutect). ^27^ for the calling of single nucleotide variants (SNVs) and small insertions and deletions (indels). ANNOVAR^28^ was then used for the annotation of the called SNVs and indels. Data processing and analysis were performed with default parameters. To estimate fusion genes, a fusion event detection tool specific for whole-exome and short-read sequencing platforms, LUMPY^29^, was used. Genes within 1000 bp each other on the same chromosome were skipped in the analysis due to their positional effect, making false matching a fusion gene.

### TCGA data processing

We analyzed somatic variant calls in the whole exome sequences of 650, 249, 209, and 42 samples for stage I, stage II, stage III, and stage IV across lung cancer cohorts (LUAD and LUSC) from The Cancer Genome Atlas (TCGA). All variants processed in a consolidated platform were obtained from the UCSC Xena Browser (http://xena.uscs.edu). Stage I and stage IV samples were analyzed as early- and late-stage samples, respectively.

### Pathway analysis

We performed a pathway analysis for the genes that were mutated and fused recurrently or non-recurrently. The Enrichr^30^ tool was used to compute enrichment p values derived from the hypergeometric distribution and adjusted for multiple testing. The pathway terms were retrieved from the KEGG^31^, Reactome^32^, Panther^33^, and Gene Ontology^34^ databases.

### Selection score analysis

The two types of selection scores were used to investigate functional implications for our identified genes that were mutated or fused. All of the two scoring methods were used to calculate selection patterns at the gene level based on the ratio of nonsynonymous to synonymous mutations across a large number of cancer patients^11,12^. However, these methods used different methodologies to compute the statistical significance of the difference between nonsynonymous and synonymous mutations, which adopted Bayesian inference^7^ or a statistical model for covariate^8^, respectively. The higher selection score derived from these scoring methods indicates stronger positive selection because a gene under positive selection will carry an extra complement of driver mutations in addition to neutral (passenger) mutations. Additionally, the statistical significance of the landscape of positive selection was tested nonparametrically by randomly selecting the same number of genes as the gene mutations (fusions) from the human genome and then comparing the mean and median of selection scores between the original list and permuted list. This permutation was repeated 1,000 times to generate a null distribution.

### Recurrence-free survival analysis

We utilized gene mutations and fusions as predictors to perform patient recurrence-free survival analysis. For our clinical trial data, the cases in which the genes were mutated or fused were classified from the controls and subjected to survival analysis. Patients who died for reasons other than cancer were excluded from the analysis. The log-rank test (Mantel-Cox test) was used to determine the significance of differences between two groups.

## Supplementary Information

Supplementary Information 1.

Supplementary Information 2.

## Data Availability

Data available on request from the authors.

## References

[1] Vogelstein B (2013). Cancer genome landscapes. Science (80-).

[2] Sasaki H (2014). Prognosis of recurrent non-small cell lung cancer following complete resection. Oncol. Lett..

[3] Soria J-C (2018). Osimertinib in untreated EGFR-mutated advanced non–small-cell lung cancer. N. Engl. J. Med..

[4] Peters S (2017). Alectinib versus crizotinib in untreated ALK -positive non–small-cell lung cancer. N. Engl. J. Med..

[5] Shaw AT (2014). Crizotinib in ROS1-rearranged non-small-cell lung cancer. N. Engl. J. Med..

[6] Planchard D (2016). Dabrafenib plus trametinib in patients with previously treated BRAFV600E-mutant metastatic non-small cell lung cancer: An open-label, multicentre phase 2 trial. Lancet Oncol..

[7] Drilon A (2018). Efficacy of larotrectinib in TRK fusion-positive cancers in adults and children. N. Engl. J. Med..

[8] Gao Q (2018). Driver fusions and their implications in the development and treatment of human cancers. Cell Rep..

[9] Ding L (2008). Somatic mutations affect key pathways in lung adenocarcinoma. Nature.

[10] Jang K, Kim K, Cho A, Lee I, Choi JK (2017). Network perturbation by recurrent regulatory variants in cancer. PLoS Comput. Biol..

[11] Weghorn D, Sunyaev S (2017). Bayesian inference of negative and positive selection in human cancers. Nat. Genet..

[12] Martincorena I (2017). Universal patterns of selection in cancer and somatic tissues. Cell.

[13] Ma J, Lyu H, Huang J, Liu B (2014). Targeting of erbB3 receptor to overcome resistance in cancer treatment. Mol. Cancer.

[14] Lee Y (2014). Role of erbB3 receptors in cancer therapeutic resistance. The unique biology of erbB3 receptors in human cancer development. Acta Biochim. Biophys. Sin..

[15] Manchado E (2016). A combinatorial strategy for treating KRAS-mutant lung cancer. Nature.

[16] Tran E (2016). T-cell transfer therapy targeting mutant KRAS in cancer. N. Engl. J. Med..

[17] Antonescu CR (2009). KDR activating mutations in human angiosarcomas are sensitive to specific kinase inhibitors. Cancer Res..

[18] Karnitz LM, Zou L (2015). Molecular pathways: Targeting ATR in cancer therapy. Clin. Cancer Res..

[19] Zighelboim I (2009). ATR mutation in endometrioid endometrial cancer is associated with poor clinical outcomes. J. Clin. Oncol..

[20] Suehara Y (2012). Identification of KIF5B-RET and GOPC-ROS1 fusions in lung adenocarcinomas through a comprehensive mRNA-based screen for tyrosine kinase fusions. Clin. Cancer Res..

[21] Zeng L, Yang N, Zhang Y (2018). GOPC-ROS1 rearrangement as an acquired resistance mechanism to osimertinib and responding to crizotinib combined treatments in lung adenocarcinoma. J. Thorac. Oncol..

[22] Sun JM, Hwang DW, Ahn JS, Ahn MJ, Park K (2013). Prognostic and predictive value of KRAS mutations in advanced non-small cell lung cancer. PLoS ONE.

[23] Ludovini V (2011). Phosphoinositide-3-kinase catalytic alpha and KRAS mutations are important predictors of resistance to therapy with epidermal growth factor receptor tyrosine kinase inhibitors in patients with advanced non-small cell lung cancer. J. Thorac. Oncol..

[24] Tuveson DA (2004). Endogenous oncogenic K-rasG12D stimulates proliferation and widespread neoplastic and developmental defects. Cancer Cell.

[25] Li H, Durbin R (2009). Fast and accurate short read alignment with Burrows-Wheeler transform. Bioinformatics.

[26] McKenna A (2010). The genome analysis toolkit: A MapReduce framework for analyzing next-generation DNA sequencing data. Genome Res..

[27] Cibulskis K (2013). Sensitive detection of somatic point mutations in impure and heterogeneous cancer samples. Nat. Biotechnol..

[28] Wang K, Li M, Hakonarson H (2010). ANNOVAR: Functional annotation of genetic variants from high-throughput sequencing data. Nucleic Acids Res..

[29] Layer RM, Chiang C, Quinlan AR, Hall IM (2014). LUMPY: A probabilistic framework for structural variant discovery. Genome Biol..

[30] Kuleshov MV (2016). Enrichr: A comprehensive gene set enrichment analysis web server 2016 update. Nucleic Acids Res..

[31] Du J (2014). KEGG-PATH: Kyoto encyclopedia of genes and genomes-based pathway analysis using a path analysis model. Mol. Biosyst..

[32] Fabregat A (2016). The reactome pathway knowledgebase. Nucleic Acids Res..

[33] Thomas PD, Campbell MJ, Kejariwal A, Mi H, Karlak B (2003). PANTHER: A library of protein families and subfamilies indexed by function. Genome Res..

[34] Blake JA (2015). Gene ontology consortium: Going forward. Nucleic Acids Res..

[35] Cerami E (2012). The cBio cancer genomics portal: An open platform for exploring multidimensional cancer genomics data. Cancer Discov..

