# Supplementary material for: Recurrence-associated gene signature in patients with stage I non-small-cell lung cancer
Source: Sci Rep. 2021 Oct 1;11:19596. doi: 10.1038/s41598-021-99197-w (PMC8486871; doi:10.1038/s41598-021-99197-w)

Supplementary Figure 1

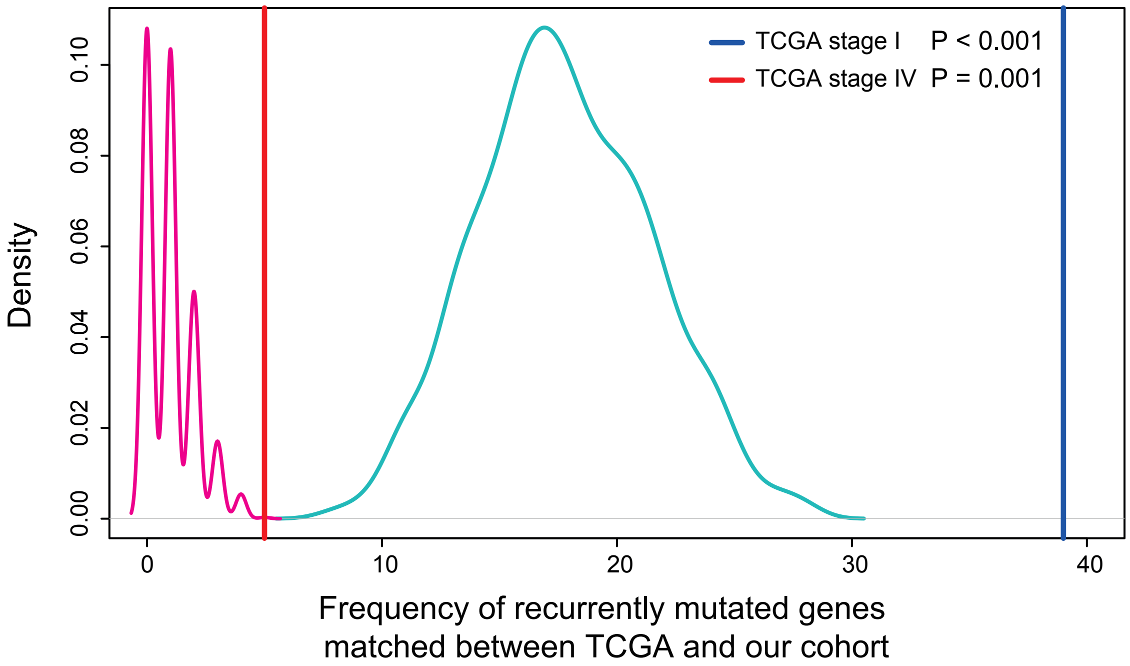

Supplementary Figure 2

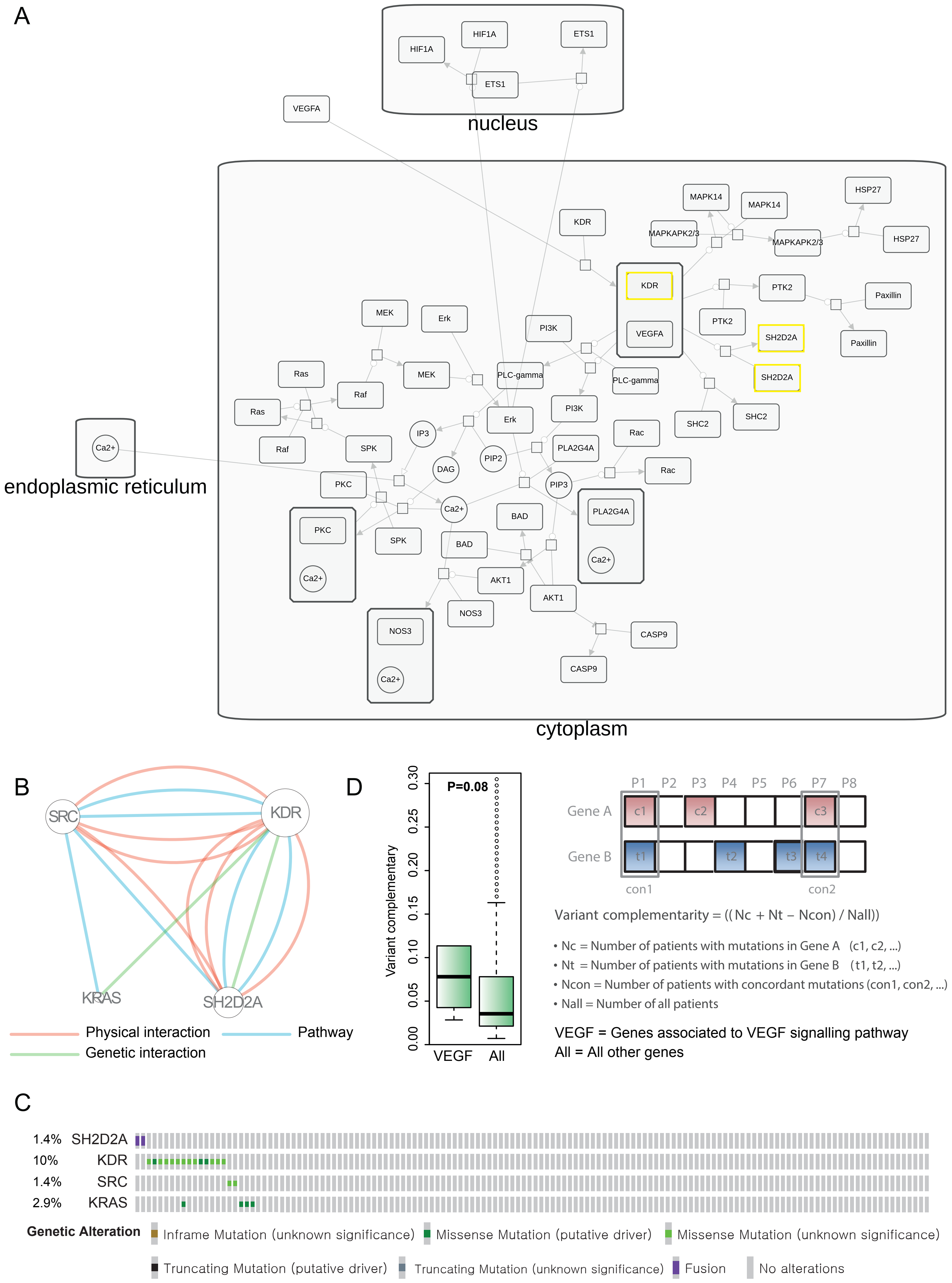

Supplementary Figure 3

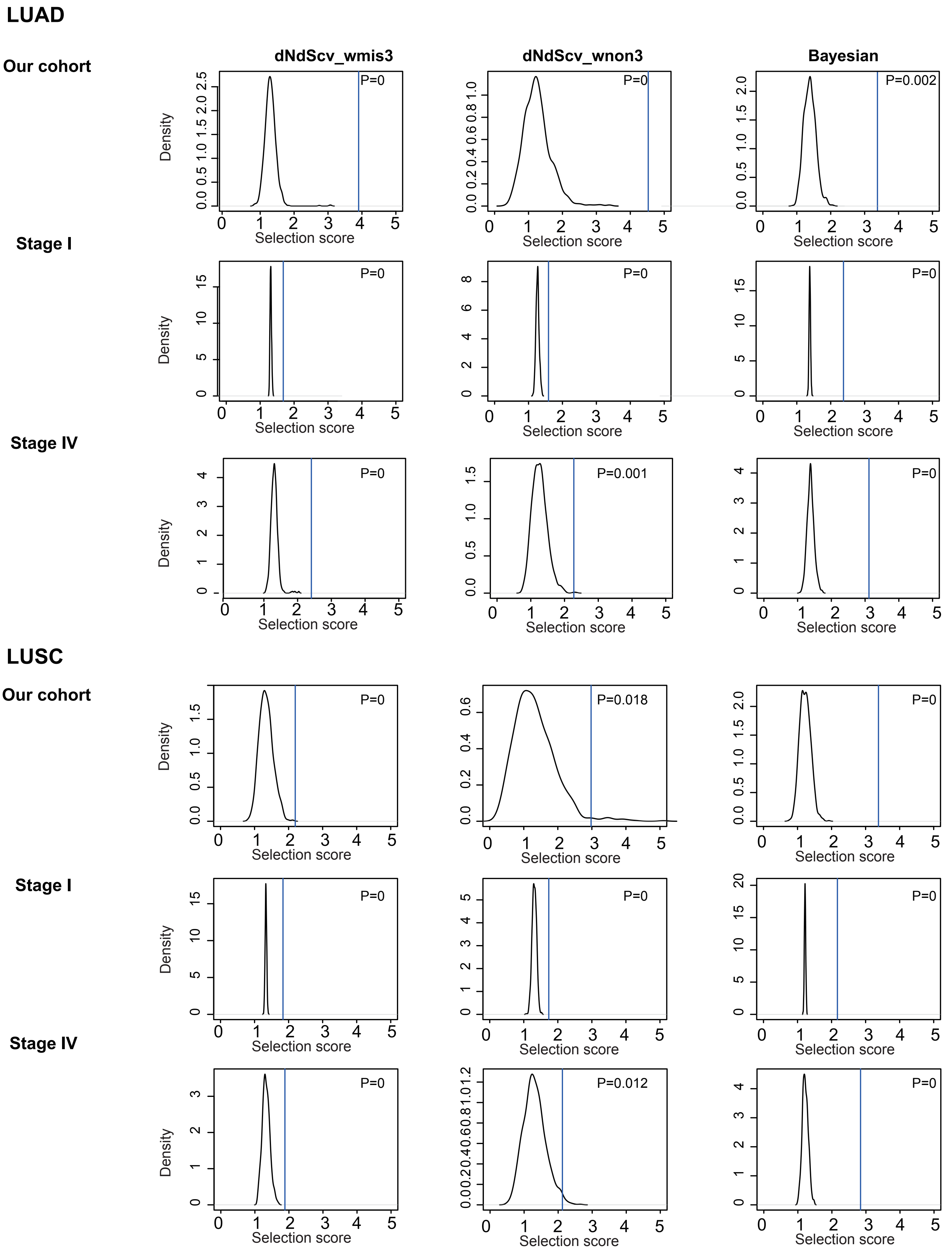

Supplementary Figure 4

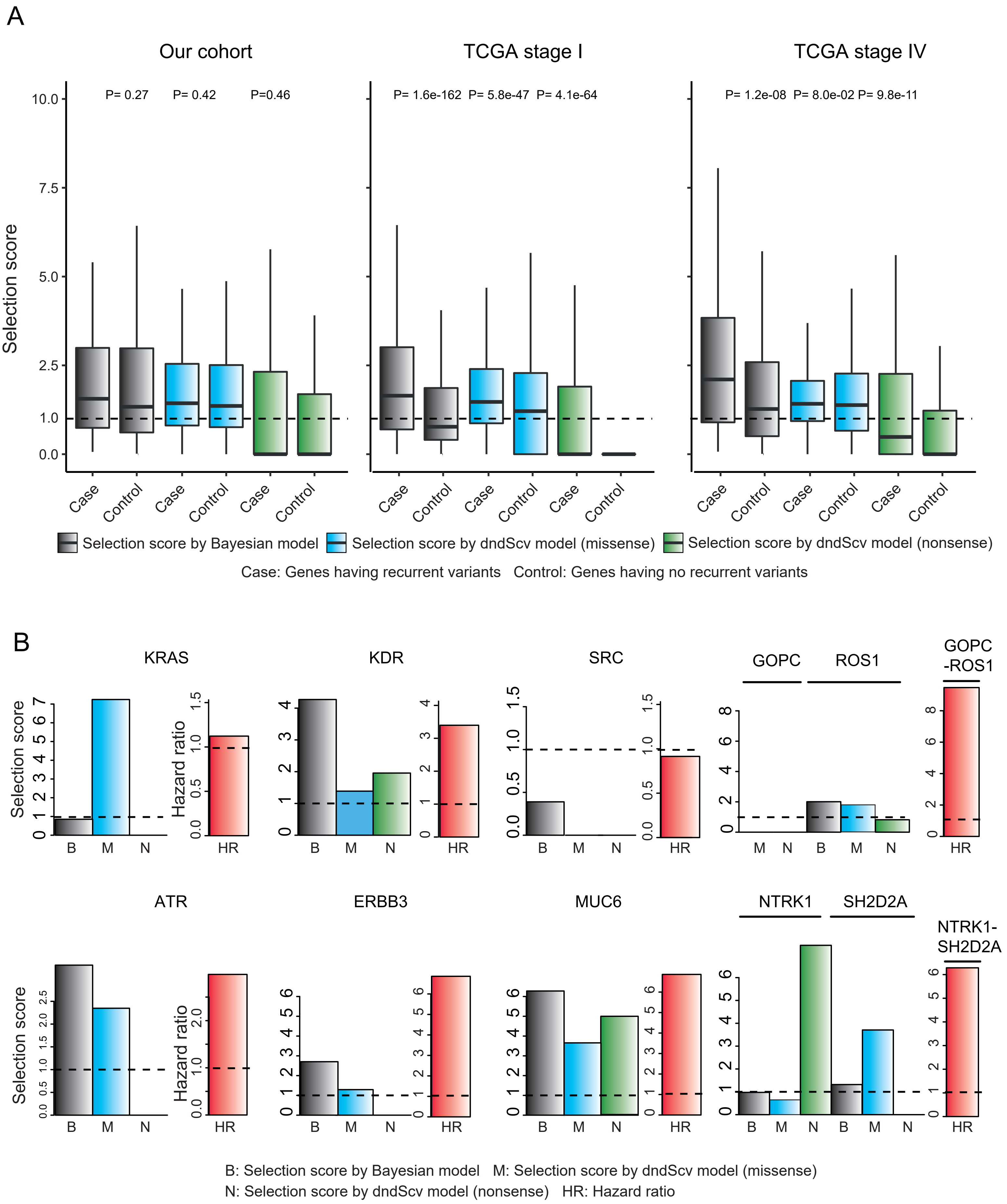

Supplementary Figure 5

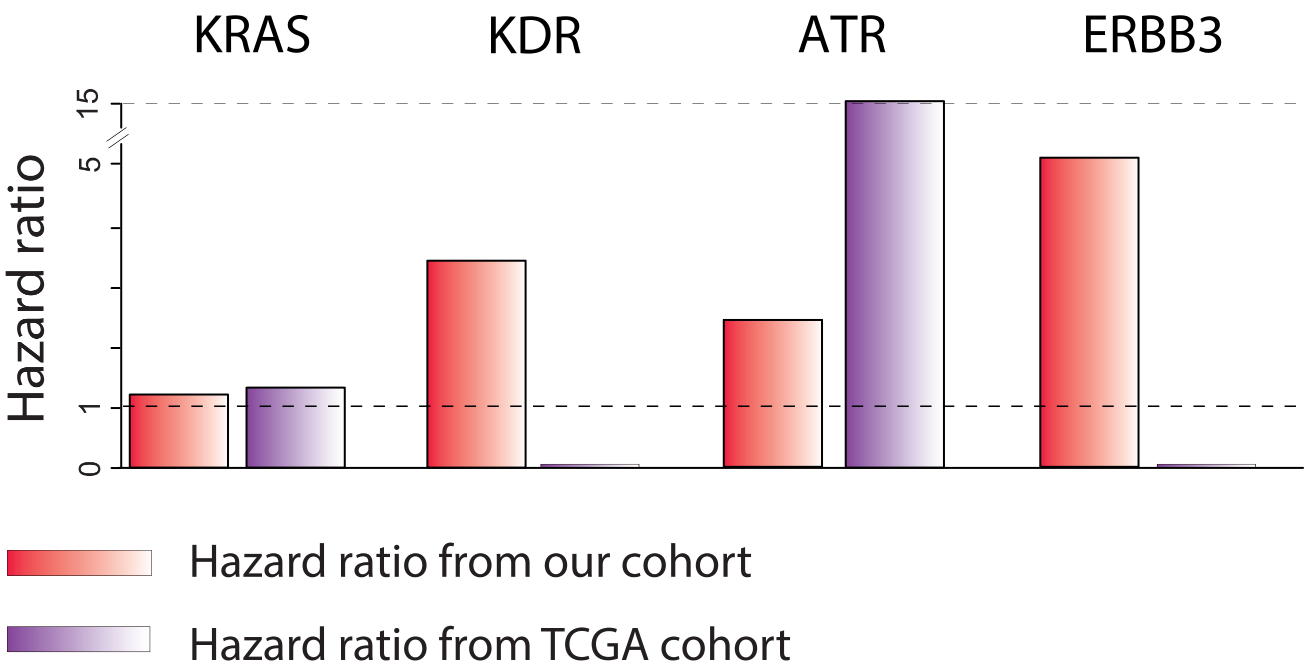

Supplement: Supplementary file 1 — Supplementary Information 1. [file 41598_2021_99197_MOESM1_ESM.pdf]
