# Supplementary material for: Recurrence-associated gene signature in patients with stage I non-small-cell lung cancer
Source: Sci Rep. 2021 Oct 1;11:19596. doi: 10.1038/s41598-021-99197-w (PMC8486871; doi:10.1038/s41598-021-99197-w)
Supplement: Supplementary file 2 — Supplementary Information 2. [file 41598_2021_99197_MOESM2_ESM.doc]

**Supplementary Figure 1. Simulation of matching the recurrently mutated genes between TCGA cohorts and our cohort**

We permutated the genes having the same number of recurrently mutated genes in our cohort to match them to the recurrently mutated genes in TCGA stage I and IV cohorts. Dark red and dark blue lines indicate the observed number of recurrently mutated genes in our cohort that matched the recurrently mutated genes in TCGA stage I patients and TCGA stage IV patients, respectively. Light red and light blue density plots show the permutation results with genes randomly chosen according to the same number of recurrently mutated genes in our cohort.

**Supplementary** **Figure 2. Systematic landscape of genes associated with the VEGF signaling pathway**

(A) The VEGF signaling pathway in a cell is described by using the PANTHER database. The genes highlighted in yellow showed a significant risk of cancer recurrence in our cohort. (B) All types of network interactions were described for the 4 genes that were identified as having a risk of cancer recurrence and enriched for the VEGF signaling pathway. (C) The landscape of genetic alterations for the 4 genes with a risk of cancer recurrence and enriched for the VEGF signaling pathway. The OncoPrinter tool^35^ was used to make this diagram by using our cohort data. (D) Description of calculating variant complementarity (left) and the result of variant complementarity for the 4 genes associated with the VEGF signaling pathway and all other genes.

**Supplementary** **Figure 3. Simulation for selective signatures of recurrently mutated genes**

The same number of recurrently mutated genes was randomly chosen (permutated) in all genes analyzed by selection score. We calculated the score of distribution for the permuted genes and how much it was different from the selection score of the recurrently mutated genes in our cohort. We also calculated the selection score for the recurrently mutated genes in TCGA early- (stage I) and late-stage (stage IV) lung cancer cohorts. Two types of selection scores derived from LUAD and LUSC were used for the simulation.

**Supplementary** **Figure 4. Selective signatures of recurrent gene mutations and fusions in an additional TCGA cohort (LUSC)**

(A) Selection values were calculated based on the Bayesian inference and covariate model (*dNdScv*) for the genes having variants of which frequency was over two. The genes with recurrent variants were compared to the genes with no recurrent variants within our cohort (left), stage I TCGA samples (middle), and stage IV TCGA samples (right). The gene numbers that were analyzed by Bayesian inference were 80, 4567, and 256 for our cohort, TCGA stage I samples, and TCGA stage IV samples, respectively. The gene numbers that were analyzed for the selection values by the covariate model were 89, 6863, and 1646 for our cohort, TCGA stage I samples, and TCGA stage IV samples, respectively. (B) Selection values for the genes that had a significant association with recurrence-free survival were described. Shown are the selection values obtained for lung squamous-cell carcinoma (LUSC) samples from TCGA. dNdScvM is the normalized ratio of nonsynonymous to synonymous mutations (dN/dS) for missense mutations.

**Supplementary** **Figure 5. Comparison of RFS analysis for the gene identified in our cohort with that in TCGA stage IV.**

The RFS analysis was performed in stage IV lung cancer patients (TCGA LUAD and LUSC) whose sample size was 42 by using the same genes that we identified as having early-stage mutations in Fig. 4B.
